# Discordance between Perceived and Actual Cancer Stage among Cancer Patients in Korea: A Nationwide Survey

**DOI:** 10.1371/journal.pone.0090483

**Published:** 2014-05-09

**Authors:** Hye-Young Shim, Jong-Hyock Park, So-Young Kim, Dong Wook Shin, Ji-Yeon Shin, Bo Young Park, Jung-Sik Huh, Hee-Young Shin, Young Joo Won, Hong Gwan Seo

**Affiliations:** 1 National Cancer Control Institute, National Cancer Center, Goyang, Korea; 2 College of Medicine/Graduate School of Health Science Business Convergence, Chungbuk National University, Cheongju-si, Korea; 3 Seoul National University Hospital and Seoul National University Cancer Hospital, Seoul, Korea; 4 Department of Preventive Medicine, School of Medicine, Eulji University, Daejeon, Korea; 5 Jeju Regional Cancer Center, Jeju, Korea; 6 Jeonnam Regional Cancer Center, Jeonnam, Korea; University of Pennsylvania, United States of America

## Abstract

**Purpose:**

We assessed the accuracy of communication between doctors and patients by evaluating the consistency between patient perception of cancer stage and the medical records, and analyzed the most influential factors of incongruence among cancer patients at 10 cancer centers across Korea.

**Methods:**

Information was gathered from cancer patients at the National Cancer Center and nine regional cancer centers located in every province of Korea between 1 July 2008 and 31 August 2008. Data were analyzed using Pearson's χ^2^ test and multivariate logistic regression analysis.

**Results:**

The stages of cancer reported by the 1,854 patients showed a low degree of congruence with the stages given in medical records (k = 0.35, *P*<0.001). Only 57.1% of the patients had accurate knowledge of their cancer stage. In total, 18.5% underestimated their stage of disease, and the more advanced the cancer stage, the more likely they were to underestimate it, in order of local (14.2%), regional (23.7%), and distant (51.6%). Logistic regression analysis showed that congruence was lower in patients with cervical cancer (odds ratio [OR] = 0.51, 95% confidence interval [CI] = 0.30–0.87), recurrence (OR = 0.64, 95% CI = 0.50–0.83), and treatment at the National Cancer Center (OR = 0.53, 95% CI = 0.39–0.72).

**Conclusion:**

There are knowledge gaps between patients' perceived and actual stage of cancer. Patients with cervical cancer, recurrence, and who received treatment at a regional cancer center showed less understanding of their cancer stage.

## Introduction

Advancements in modern medicine have increased cancer survival rates, and cancer is now considered a curable disease. Patients are actively involved in making decisions about their care, resulting in improved quality of life [1].

Patients' knowledge influences their ability to actively participate in decision-making regarding their medical care and treatment choices as well as their ability to manage their condition and thus improve their own medical outcome [2], [3]. Patients should have accurate knowledge of their own disease state to make educated treatment decisions and be actively engaged in decision-making processes [4]. In a previous study, the all-cause and cancer-specific survival of a cancer-status-aware group was 1.3-fold higher than that of a non-aware group [5]. Additionally, a group of patients who were aware of having incurable cancer showed a higher health-related quality of life than patients who were unaware of that status [6].

However, in a recent study in Korea, although a majority of patients wanted to be fully informed about their diagnosis, cancer stage, prognosis, and treatment plan (73.8%), only 33.3% of them were provided with sufficient information [7]. In another study, 86% of cancer patients knew that they had cancer, but merely 37% had accurate knowledge of their stage [8]. In an USA study, 98% of cancer patients knew their medical diagnosis and 91% knew their cancer location, whereas only 25% knew their cancer stage [9]. In particular, older, low-income, and male patients showed less understanding of their cancer stage.

Identifying cancer stage provides a basis for predicting survival, choosing an initial treatment, establishing accurate communication among healthcare providers, and reporting outcomes in a uniform manner [10]. In particular, the cancer stage is an indicator of the progression of cancer and plays a very important role in determining the goals of patient care. Therefore, it is crucial for patients to have accurate knowledge about their cancer stage for shared decision-making regarding their care [5], [11].

We evaluated the accuracy of patients' knowledge about their cancer stage and examined the factors that influence this knowledge.

## Materials and Methods

### Study design and population

Information was gathered from cancer patients at the National Cancer Center and nine regional cancer centers, one in each Korean province, from 1 July to 31 August 2008. Quota sampling was used: 80% of the patients had been diagnosed with one of six major types of cancer (stomach, lung, liver, colon and rectum, breast, or cervix), and the remaining 20% had other types. The inclusion criteria were age of >18 years, an established diagnosis of cancer, a period of >4 months since diagnosis, current treatment or follow-up, and written informed consent for study participation. Over a period of 2 months, cancer patients who agreed to participate were interviewed at their treatment centers by trained interviewers. At each of the 10 cancer centers, outpatients were recruited while visiting the department, and inpatients were visited in the ward by the investigators. The detailed procedures have been described elsewhere [12]. Before this study, pilot surveys were conducted at each of the cancer centers using the survey methods employed in this study. No problems were found with respect to patients understanding the questions or with the content validity of the questionnaires. The National Cancer Center Institutional Review Board approved the study.

**Figure 1:**
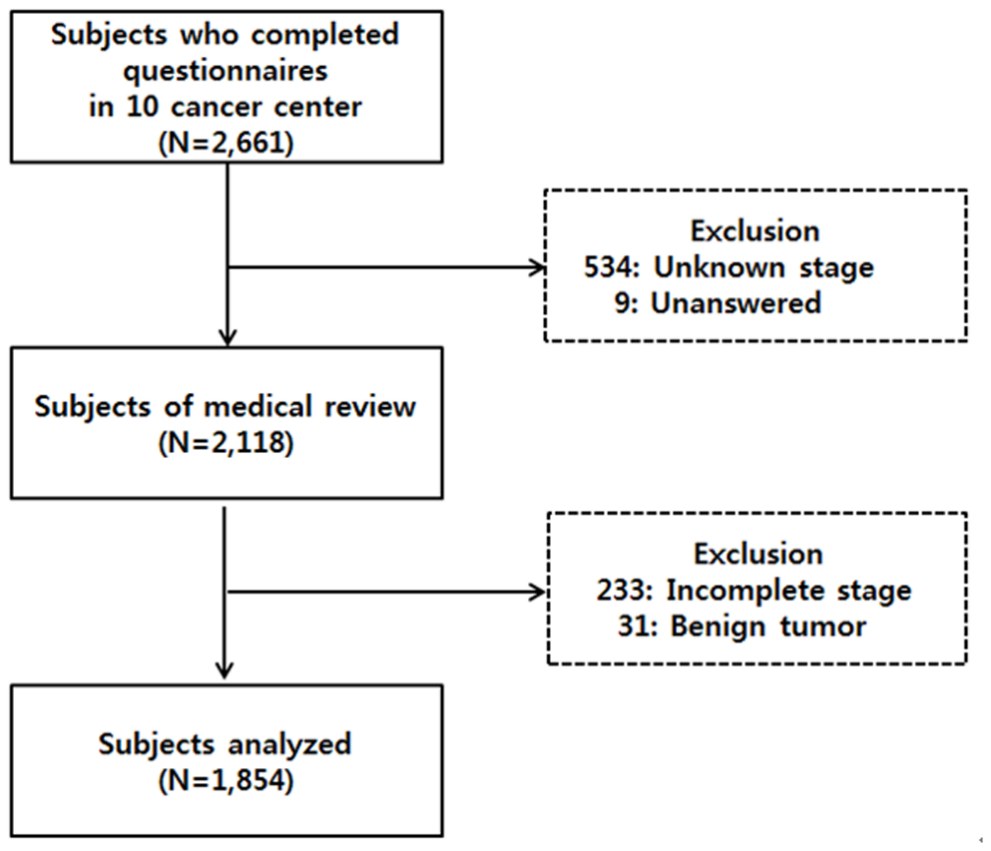
Flow chart of study population.

In total, 2,661 cancer patients completed an interview. Of these, 534 were excluded because they did not know their cancer stage and 9 were excluded because they gave no answers. Medical records were for 2,118 patients with regard to their cancer stage; 233 were excluded due to incomplete information that precluded determination of their cancer stage, and 31 were excluded because they had benign tumors. As a result, the final analysis was conducted on the records of 1,854 patients (Figure 1).

Through a retrospective analysis of the patients' medical records, we obtained clinical data such as cancer type, histology, and the Surveillance Epidemiology and End Results (SEER) summary stage [13].

### Measurements

In the questionnaire survey, the patients received four pages of questions. The survey included questions about clinical information and socioeconomic variables. Regarding the clinical information, the question regarding the stage of cancer was, “Did you know the stage of your cancer at diagnosis?” and the response options were stage 0, stage 1, stage 2, stage 3, stage 4, and unknown. Furthermore, the site of the cancer (stomach, lung, liver, colon, breast, cervix, others), recurrence (yes/no), treatment (surgery, chemotherapy, radiotherapy, other), institution type (National Cancer Center, regional cancer center), and quality of life (no problem, some problems, severe problems) were surveyed. Quality of life was measured using the EQ-5D questionnaire, which measures five dimensions of quality of life (mobility, self-care, usual activities, pain/discomfort, and anxiety/depression) using a three-point scoring system (1 =  no problem, 2 =  some problems, and 3 =  severe problems). The EQ-5D has been validated for Korean subjects [14]. We classified responses to the EQ-5D into two categories: patients with at least one problem, and those with no problem. sociodemographic factors included gender (male, female), age (≤39, 40–59, 60–69, ≥70 years), education (≤middle school, high school, ≥college), marital status (single, married, separated/divorced/widowed), income (<2000, 2000–3999, ≥4000 USD), and occupation (housewife, office worker, non-office worker, service/manufacturing worker, farmer/forest worker/fisherman, none).

After completion of the survey, medical chart audits were performed by health record administrators to determine the origin of the cancer, recurrence, date of cancer diagnosis, type of treatment, metastasis, and stage of cancer at diagnosis using the SEER summary stage system. The survey results were subsequently checked against the patient's medical records for accuracy, and graded accordingly. Patient names were coded during the analysis to maintain anonymity.

### Statistical analysis

We compared the general characteristics between the congruent and incongruent groups using the chi-square test, selected demographics (sex, age, education, marital status, income, and occupation), and clinical variables (cancer site, recurrence, SEER summary staging, treatment, institutional type, and quality of life). In addition, we used Cohen's kappa statistic to assess agreement between the disease stages reported on the questionnaire and those found in relevant medical records.

We conducted multiple logistic regression to identify the factors associated with incongruence. In the multivariable model, basic sociodemographic factors such as age, sex, education, marital status, income, and SEER stage of clinical factors were incorporated as covariates regardless of the results of bivariate analysis, and other variables with a *P* value of <0.05 in univariate analysis were included in the model as potential predictors. The variables were cancer site, recurrence, treatment, and institution type. Data from patients with missing values were excluded from the multiple logistic regression models. All statistical analyses were conducted using SPSS version 12.0 (SPSS, Inc., Chicago, IL). Statistical significance was set at a two-sided *P* value of <0.05.

We conducted multiple logistic regression to identify factors associated with incongruence. In the multivariable model, basic sociodemographic factors, such as age, gender, education, marital status, income, and SEER stage of clinical factors, were incorporated as covariates regardless of the results of the bivariate analysis, and other variables with a *P* value of <0.05 in univariate analysis were included in the model as potential predictors. Those variables were cancer site, recurrence, treatment, and institution type. Data from patients with missing values were excluded from the multiple logistic regression models. All statistical analyses were conducted using the SPSS software (ver. 12.0; SPSS, Inc., Chicago, IL). Statistical significance was set at a two-sided *P* value of <0.05.

## Results

To compare the general characteristics of the congruent and incongruent groups, a chi-squared test was conducted to check the differences in distribution. Differences were found in marital status, cancer site, recurrence, SEER stage, and institutional type. In the congruent group, the rates of congruence were high for the factors married (86.9%), stomach cancer (22.2%), no recurrence (83.8%), regional stage (46.6%), and regional cancer center (84.7%); in the incongruent group, they were high for the factors married (82.4%), cancer at other sites (23.0%), no recurrence (86.3%), localized stage (46.3%), and regional cancer center (91.6%) (Table 1).

**Table 1:** Characteristics of the congruent and incongruent groups.

Variable	Congruent (n = 1059)	Incongruent (n = 795)	Total	*P* (χ^2^ test)
	n (%)	n (%)	n (%)	
Sex				
Male	514 (48.5)	417 (52.5)	931 (50.2)	0.10
Female	545 (51.5)	378 (47.5)	923 (49.8)	
Age in years				
≤39	67 (6.3)	52 (6.5)	119 (6.4)	0.06
40–59	498 (47.0)	364 (45.8)	862 (46.5)	
60–69	330 (31.2)	220 (27.7)	550 (29.7)	
≥70	164 (15.5)	159 (20.0)	323 (17.4)	
Education				
≤Middle school	543 (51.5)	397 (49.9)	940 (50.8)	0.69
High school	337 (31.9)	255 (32.1)	592 (32.0)	
≥College	175 (16.6)	143 (18.0)	318 (17.2)	
Marital status				
Single	22 (2.1)	26 (3.3)	48 (2.6)	0.02
Married	919 (86.9)	655 (82.4)	1,574 (84.9)	
Separated/divorced/widowed	117 (11.1)	114 (14.3)	231 (12.5)	
Income in USD				
<2000	548 (52.0)	430 (54.4)	978 (53.0)	0.60
2000–3999	347 (33.0)	246 (31.1)	593 (32.2)	
≥4000	158 (15.0)	115 (14.5)	273 (14.8)	
Occupation				
Housewife	242 (24.1)	180 (23.5)	422 (23.8)	0.14
Office worker	101 (10.0)	94 (12.3)	195 (11.0)	
Non-office worker	229 (22.8)	185 (24.2)	414 (23.4)	
Service, manufacturing worker	80 (8.0)	58 (7.6)	138 (7.8)	
Farmer, forest worker, fisherman	199 (19.8)	117 (15.3)	316 (17.8)	
None	155 (15.4)	131 (17.1)	286 (16.1)	
Cancer site				
Stomach	235 (22.2)	138 (17.4)	373 (20.1)	<0.001
Lung	145 (13.7)	113 (14.2)	258 (13.9)	
Liver	59 (5.6)	69 (8.7)	128 (6.9)	
Colon	165 (15.6)	110 (13.8)	275 (14.8)	
Breast	209 (19.7)	130 (16.4)	339 (18.3)	
Cervix	47 (4.4)	52 (6.5)	99 (5.3)	
Others	199 (18.8)	183 (23.0)	382 (20.6)	
Recurrence				
Yes	169 (16.2)	184 (23.7)	353 (19.4)	<0.001
No	873 (83.8)	593 (76.3)	1466 (80.6)	
SEER				
*In situ*	5 (0.50)	4 (0.50)	9 (0.50)	<0.001
Localized	399 (37.7)	368(46.3)	767(41.4)	
Regional	494 (46.6)	258 (32.5)	752 (40.6)	
Distant	161 (15.2)	165 (20.8)	326 (17.6)	
Treatment				
Surgery	251 (24.2)	183 (23.8)	434 (24.0)	0.22
Chemotherapy	107 (10.3)	68 (8.9)	175 (9.7)	
Radiotherapy	12 (1.2)	19 (2.5)	31 (1.7)	
Surgery + Chemotherapy	341 (32.9)	233 (30.3)	574 (31.8)	
Surgery + Radiotherapy	55 (5.3)	49 (6.4)	104 (5.8)	
Chemotherapy + Radiotherapy	68 (6.6)	59 (7.7)	127 (7.0)	
Surgery + Chemotherapy + Radiotherapy	204 (19.7)	157 (20.4)	361 (20.0)	
Institution type				
National Cancer Center	162 (15.3)	67 (8.4)	229 (12.4)	<0.001
Regional cancer center	897 (84.7)	728 (91.6)	1,625 (87.6)	
EQ-5D score				
Normal	673 (63.6)	516 (64.9)	1,189 (64.1)	0.55
Disability	386 (36.4)	279 (35.1)	665 (35.9)	

An analysis of the agreement between the perceived cancer stage and the cancer stage found in the medical records revealed a Cohen's kappa of 0.35 (*P*<0.001). Only 57.1% of all patients had accurate knowledge of their cancer stage. By stage, the agreement rate was *in situ* (55.6%), local (52.0%), regional (65.7%), and distant (49.4%). The percentage of patients who underestimated their stage was 18.5%. The more advanced the disease, the more likely the patients were to underestimate it, in the order of local (14.2%) <regional (23.7%)<distant (51.6%) stage (Table 2).

**Table 2:** Comparison of perceived and actual stages of cancer.

Perceived stage(n = 1854)	Actual stage (n = 1854)					Weighted kappa	*P* value
	*in situ*	Local	Regional	Distant	Sum		
*In situ*	5 (55.6)	109 (14.2)	36 (4.8)	6 (1.8)	156 (8.4)	0.35	<0.001
Local	3 (33.3)	399 (52.0)	142 (18.9)	30 (9.2)	574 (31.0)		
Regional	0 (0.0)	227 (29.6)	494 (65.7)	129 (39.6)	850 (45.8)		
Distant	1 (11.1)	32 (4.2)	80 (10.6)	161 (49.4)	274 (14.8)		
Sum	9 (100.0)	767 (100.0)	752 (100.0)	326 (100.0)	1854 (100.0)		
Agreement (p/a)	55.6%	52.0%	65.7%	49.4%	57.1%		
Underestimation	-	14.2%	23.7%	51.6%	18.5%		
Overestimation	44.4%	33.8%	10.6%	-	24.4%		

Univariate analysis revealed that the following factors significantly influenced a low awareness of cancer stage (*P*<0.05): cancer type, recurrence, treatment method, institution type, and socio-demographic variables such as age, sex, education, marriage status, income, and SEER stage. These factors were incorporated into a multivariate logistic regression analysis. The factors that affected incongruence were cancer type, recurrence, and institution type. In terms of cancer type, cervical cancer (OR = 0.51, 95% CI = 0.30–0.87) had a weaker effect on incongruence than did stomach cancer. In terms of recurrence, the presence of recurrence (OR = 0.64, 95% CI = 0.50–0.83) had a weaker effect on incongruence than did the absence of recurrence. In terms of institution type, regional care centers (OR = 0.53, 95% CI = 0.39–0.72) had a stronger effect on incongruence than did the National Care Center (Table 3).

**Table 3:** Factors associated with incongruent between perceived and actual cancer stage.

Variable	Adjusted OR*	95% CI†
Sex		
Male	1.00	Reference
Female	1.24	0.96–1.60
Age		
≤39	1.00	Reference
40–59	0.90	0.59–1.39
60–69	0.96	0.60–1.56
≥70	0.62	0.37–1.03
Education		
<Middle school	1.00	Reference
High school	0.93	0.73–1.18
≥College	0.85	0.62–1.16
Marital status		
Single	1.00	Reference
Married	1.60	0.85–3.01
Separated/divorced/widowed	1.19	0.60–2.38
Income in USD		
<2000	1.00	Reference
2000–3999	0.950	0.75–1.21
≥4000	0.979	0.71–1.35
Cancer site		
Stomach	1.00	Reference
Lung	0.77	0.52–1.14
Liver	0.67	0.41–1.09
Colon	0.90	0.67–1.27
Breast	0.85	0.57–1.28
Cervix	0.51	0.30–0.87
Other	0.72	0.52–1.00
Recurrence		
No	1.00	Reference
Yes	0.64	0.50–0.83
Treatment		
Surgery	1.00	Reference
Chemotherapy	1.44	0.93–2.23
Radiotherapy	0.60	0.27–1.34
Surgery + Chemotherapy	0.98	0.73–1.31
Surgery + Radiotherapy	0.89	0.56–1.43
Chemotherapy + Radiotherapy	0.97	0.61–1.57
Surgery + Chemotherapy + Radiotherapy	0.91	0.64–1.30
Institution type		
National Cancer Center	1.00	Reference
Regional cancer center	0.53	0.39–0.72
SEER stage		
*In situ*	1.00	Reference
Localized	0.83	0.29–3.19
Regional	1.39	0.36–5.34
Distant	0.65	0.17–2.58

^*^Multivariate analysis was adjusted for sex, age, education, marital status, income, cancer site, recurrence, treatment, institution type, and SEER stage.

† 95% CI: 95% confidence interval.

The *P* value for the Hosmer–Lemeshow test was 0.36. This indicates an adequate ability to distinguish between groups, and there was no evidence of a lack of fit regarding the model tested.

## Discussion

To the best of our knowledge, this is the first nationwide comparison of data obtained through face-to-face interviews regarding patients' perceived cancer stage versus their actual stage obtained from their medical records. Accurate information about disease and treatment can help patients to cope with uncertain situations and manage their disease rationally. However, only 57.1% of patients had congruence between their perceived and actual stage of cancer. In particular, half of the patients with an advanced stage underestimated their stage. This implies that the more advanced the stage, the less likely patients are to have accurate knowledge of their disease status. Various factors affected the patients' insufficient knowledge about their stage.

First, physician-associated factors may influence patients' awareness of their disease stage. Doctors may be less likely to provide patients with detailed information about advanced-stage cancer out of a fear to lose the patient's confidence and trust [15]. Furthermore, doctors' disclosure of disease information varies greatly by country. In 2002, a survey was conducted among cancer specialists who participated in a meeting of the American Society of Clinical Oncology. In total, 17% of North American physicians responded that in cases of cancer with a poor prognosis, they would not inform patients of their status, compared with 33% in non-North American countries, including southern Europe, the Middle East, Asia, and Latin America [16]. In the East, including Korea, and in southern and eastern Europe, physicians tend not to keep patients informed, based on the concept that such disclosure would not aid in the patient's healthcare [17]–[19]. In a Chinese study, there was a tendency among physicians not to give bad news because if patients were informed of their diagnosis, they would lose hope, feel frustrated, and give up fighting their cancer [20], [21]. In a Chinese study of oncology clinicians, 87.5% reported that patients with early-stage cancer should be informed of the diagnosis, while only 40.5% believed that patients with terminal illness should be informed [21]. In North America, the percentage of disclosure is high because promotion of patient autonomy to ensure the right of an individual to make his or her own healthcare decisions is in accordance with Western ethical values and legal norms [22], [23]. Among Eastern cultures, Japan has established constitutional guidelines for cancer diagnosis disclosure to ensure that cancer patients are directly informed of their diagnosis [24] as part of the efforts to keep patients accurately informed about their disease.

Meanwhile, from a patient's point of view, the low degree of agreement between the perceived stage and the actual stage might be affected by the patient's own passive attitude toward requiring information. This tendency is reportedly present among patients with advanced-stage cancer. In one previous study, when the hypothetical diagnosis shifted from early-stage cancer to terminal illness, patients with terminal-stage cancer who wanted to be informed about their diagnosis declined significantly from 90.8% of those with early-stage cancer to 60.5% of those with terminal-stage cancer [25]. In Korea, patients with terminal-stage cancer also showed a lower disclosure preference than did those with early-stage cancer [26]. Even among countries where honesty is commonplace, patients may be reluctant to be fully informed of their diagnosis or prognosis [27]. Because a patient's preference for disclosure of their disease status varies depending on the individual's values and beliefs, these results emphasize the importance of informing individuals based on their preference, regardless of sex, age, type of cancer, cultural background, or other factors [28].

Family also has a significant impact on patient awareness of disease information. In Eastern countries, including Korea, patients' families tend be informed first and in more detail about patients' disease status [23], [29]. One Japanese study found that 22.5% of physicians informed patients directly about their disease status and treatment plan, while 98.1% tended to inform patients' families first[30]. In Taiwan, 62.6% of physicians preferred to inform the relatives [31]. In a Singaporean study, about 84% of physicians did not inform patients of their cancer diagnosis at the request of the patients' families [32]. In a Korean study, 78% of cancer patients informed their families of the terminal-stage status first, while only 26% of the patients were informed [8]. In a 2013 study, patients' guardians were informed in 32.3% of cases, showing that the family plays an important role in decision-making [7]. However, due to social norms and values in the US, 97% of patients prefer their doctors to directly inform them of their diagnosis; this is a higher rate than in Eastern countries [22], [33]. In 1998, Japan legislated diagnosis discourse guidelines that required doctors to inform patients of their diagnosis before their families and relatives to allow patients to obtain accurate knowledge about their disease [24]. Because the results of the present study showed that merely 57.1% of patients were aware of their cancer stage, Korea should establish an institutional strategy similar to that in Japan so that doctors are required to inform patients of their disease status before their families.

There is also a possibility that the structure of the Korean cancer care system might have affected the low degree of congruence. Under the Korean system, most patients are diagnosed and treated at tertiary-level institutions. It is common for oncologists to see 20–60 patients per session over 3–4 h [34]. Clearly, this means that physicians have little time to address patients' concerns. In Korea, the average patient with cancer spends 7.0 min with their physician, which is shorter than the amount of time primary-care physicians spend in countries such as the Netherlands (10.2 min), Belgium (15.0 min), and Switzerland (15.6 min) [34]. Thus, limitations of the care system and the average duration of a physician visit are among the factors that influence patients' insufficient knowledge of their treatment.

We also analyzed factors that affect patients' inaccurate knowledge of their cancer stage. Among cancer types, the cervical cancer group, the recurrence group, and the regional cancer center group had particularly inaccurate knowledge of their cancer stage.

The results for cervical cancer may be explained by the fact that it is a sexually transmitted disease, and both physicians and patients tend to be reluctant to engage in discussions about sex. Many patients reportedly lack information or knowledge about this subject, have a conservative attitude towards sex, and/or feel ashamed of and resistant to reproductive treatments [35]. With respect to cancers of females, Korean society tends to have androcentric views and assumptions, entrenched modesty issues, and victim-blaming tendencies [36]. Moreover, with respect to cervical cancer, female patients may encounter barriers in obtaining accurate information about their disease because of the cultural prejudices of Korean society.

Our results indicate that patients who experience recurrence are often unaware of their cancer stage. Most patients with recurrent cancer still face significant difficulties in that they are not informed when their disease progresses to its terminal phase and when treatment transitions from curative to palliative [37]. Some studies have suggested that patients with recurrence experience more difficulties in terms of health perceptions, physical function, somatization, and effects on life and medical interactions than do newly diagnosed patients, those undergoing adjuvant therapy, and those in a stable phase of their disease [38]. Many patients who develop recurrence express dissatisfaction with their initial choice of treatment, tend to blame themselves, and distrust their families and medical professionals [39]. As in previous studies, we also estimated that patients with recurrent cancer have a lower awareness of their cancer stage compared with when they were first diagnosed.

In terms of institutional characteristics, regional cancer centers showed less agreement than did the National Cancer Center. Physicians' lack of training may be among the reasons for a lower rate of disclosure of disease information to patients [40] and physicians may require certain levels of communication skills, emotional capability, training, and experience to be able to deliver bad news [41]. In many countries, inadequate training is provided for medical students and physicians about how to “break the bad news” to patients. Despite the lack of active training in other countries [42], [43], the Korea National Cancer Center provides training on communication skills as a 16-h mandatory course for medical staff (physicians, nurses, and other healthcare professionals) three times per year. However, regional cancer centers do not yet have such training programs in place. This seems to influence more strongly the lower awareness of cancer stage among patients at regional cancer centers than among those at the National Cancer Center. Such training for physicians should be expanded to regional cancer centers, and future studies should evaluate the effectiveness of this training.

This study has several limitations. First, patient data were collected at the National Cancer Center and nine regional cancer centers, one in each of the provinces of Korea. Korean cancer patients are able to select a cancer center or another hospital for treatment. Therefore, although we selected a regional cancer center in each province of Korea, our patient sample may not be truly representative because of the participant-selection method. Second, we were unable to perform random sampling at any of the hospitals. We could only obtain information from those who agreed to be interviewed. Third, doctor characteristics (such as age, sex, training/knowledge, attitudes, and behaviors) may affect patient–doctor communication [44]. However, we did not include an analysis of such factors; therefore, this requires further consideration.

In conclusion, there is incongruence between patients' perceived and actual cancer stage and we identified some factors that influence this incongruence. The more advanced the cancer, the less aware patients tend to be about their cancer stage. In addition, incongruence tends to be most associated with cervical cancer, recurrence, and treatment at regional cancer centers. Our study provides useful basic data for the establishment of guidelines for diagnosis disclosure to overcome barriers in communication between physicians and patients, by taking into account factors that lead to lower awareness of disease information, promoting communication skills training for physicians, and reorganizing inadequate or absent communication systems between physicians and patients.
